# Dietary supplementation with xylanase suppresses the antinutritional effect of nonstarch polysaccharides of flaxseed and increases bone strength in broiler chickens

**DOI:** 10.1371/journal.pone.0312950

**Published:** 2024-11-01

**Authors:** Miloš Skřivan, Michaela Englmaierová, Milan Marounek, Tomáš Taubner, Davide Lanzoni, Klára Bejčková, Carlotta Giromini, Antonella Baldi

**Affiliations:** 1 Department of Nutritional Physiology and Animal Product Quality, Institute of Animal Science, Uhrineves, Prague, Czech Republic; 2 Department of Veterinary Medicine and Animal Sciences, University of Milan, Milan, Italy; 3 Coordinating Research Centres, Innovation for Well-Being and Environment, University of Milan, Milan, Italy; Zagazig University Faculty of Agriculture, EGYPT

## Abstract

The aim of this study was to determine the effects of xylanase and flaxseed the performance of chickens, digesta viscosity, nutrient retention, fatty acid profile in muscle, tibia strength and interrelations of these factors in broiler chickens fed a wheat-based diet. Seven hundred and twenty one-day-old Ross 308 cockerels were assigned to four treatments according to the contents of flaxseed (0 and 80 g/kg) and xylanase (0 and 0.1 g/kg) in the diet. Xylanase significantly decreased the intake of feed (p < 0.001), decreased feed conversion (p < 0.001), and reduced mortality (p = 0.050). In addition, xylanase significantly increased the retention of all nutrients (p = 0.010 –<0.001) except crude fibre, the fat content in breast meat (p = 0.029) and liver (p = 0.019) and the concentration of polyunsaturated fatty acids (PUFAs) in meat (p = 0.002). Flaxseed supplementation did not influence performance but decreased the retention of dry matter (p = 0.016), crude protein (p = 0.012), organic matter (p = 0.016) and nitrogen-free extract (p = 0.008). Xylanase in combination with flaxseed increased the content of n-3 fatty acids in the breast meat (p = 0.006). The lowest n-6/n-3 ratio (p = 0.001) was detected in the flaxseed and flaxseed combined with xylanase groups. Significant interaction effects of flaxseed and xylanase on tibia strength (p = 0.030) and tibia ash content (p = 0.009) were detected. The administration of xylanase or flaxseed alone increased tibia strength. Compared with the control diet, the addition of flaxseed to the diet increased the digesta viscosity (p = 0.043) in the ileum, whereas the addition of xylanase decreased the level of this indicator. It can be concluded that xylanase is an enzyme suitable for increasing nutrient availability, and in the case of its addition to a flaxseed diet, it can reduce the antinutritional effect of flaxseed by reducing the viscosity of the digesta and increasing the content of health-promoting n-3 PUFAs.

## Introduction

Meat types of poultry have long been selected for their intensity of growth and conversion of feed, although this selection process leads to a shorter duration of broiler chicken breeding and more problems with bone formation [1]. Nevertheless, there are precautions in the field of nutrition for improving the skeletal health of poultry [2, 3]. Dietary polyunsaturated fatty acids (PUFAs) with an n-6/n-3 ratio of 1.0 resulting from the addition of α-linolenic acid (ALA, 18:3) increased the strength of the sternum of hens by 40–60% [4]. However, increased dietary levels of PUFAs with C20–C22 long carbon chains reduced bone strength [5]. Nevertheless, Ao et al. (2015) [6] reported a significantly greater strength of the humerus in broiler chickens fed a diet supplemented with 1% or 2% docosahexaenoic acid (DHA). Additionally, measurement of the ash content in bones, an indicator of bone strength, in hens fed a diet supplemented with n-6 PUFAs did not yield consistent results. Liu et al. (2003) [7] reported a positive relationship, whereas Baird et al. (2008) [8] reported no relationship. Thanabalan et al. (2022) [9] presented a possible cause for the different results, as they showed in their meta-analysis that increasing dietary values of the n-6 to n-3 PUFA ratio are accompanied by a decreasing content of bone ash in meat chickens. They developed a model for the prediction of tibial bone ash content (BAC) based on PUFA and calcium (Ca) concentrations: BAC = 44.01–0.25 × (dietary n-6/n-3)– 0.07 × Ca (%). According to the authors, the enrichment of feed for broilers with n-3 PUFAs may reduce the incidence of skeletal disorders. The effect of the Ca content in the diets from starter to finisher was only 0.1%, and the effect of Ca was not significant. A diet for broiler chickens containing more than 50% of linoleic acid (LA) from total PUFAs acts as a pro-inflammatory agent [10]. Cytokines, e.g., interleukin 6 and prostaglandin E_2,_ participate together with increased n-6 PUFAs in weakening bone strength and contributing to the loss of bone mass [7].

Flaxseeds are rich in ALA. The presence of ALA in the diets of chickens increases the content of this essential fatty acid (FA) in tissues [11, 12]. Flaxseeds are also a deposit of antinutrients, soluble and insoluble nonstarch polysaccharides (NSPs), mucin and cyanogenic glycosides, which decrease the digestibility of nutrients [13]. The high content of dietary fibre or NSPs in flaxseeds may have a negative effect on the growth performance of animals, especially chickens [14–16]. More specifically, the soluble component present in the hull fraction is able to dissolve in water, forming an intestinal environment characterised by high viscosity [17–19], consequently limiting digestion and nutrient absorption in chickens [20]. The antinutritional properties of NPSs, however, can be mitigated by the supplementation of enzyme preparations containing xylanase [21, 22]. As reported by Baker et al. (2021) [23], the response to xylanase supplementation within the diet may be a reduction in digesta viscosity, increased nutrient release from polysaccharides or a combination of these factors. Jia et al. (2009) [24] and Apperson and Cherian (2017) [25] reported positive effects of carbohydrases on the concentrations of ALA and PUFA n-3 in the bodies of flaxseed-fed chickens. Head et al. (2019) [26] extended this research and studied the effect of a mixture of 7 carbohydrases, including xylanase and 10% flaxseed, on hepatic PUFA n-3 levels and the expression of enzymes involved in lipid metabolism. Supplementation with enzymes eliminated the negative effect of NSPs in flaxseed and positively influenced the content of lipids and the expression of genes involved in lipid metabolism in the hepatic tissues of broilers.

In poultry farming, wheat is the main source of energy because its price is lower and more stable than that of corn. In mixed feeds for chickens, it is represented by up to 50%. Wheat, however, is also characterised by the presence of antinutritional factors, particularly NSPs. Antinutritional factors are able to resist animal digestive enzymes and consequently create a highly viscous intestinal environment, impairing chick growth [27]. More specifically, the main NSPs are water-soluble arabinoxylans, β-glucans, xyloglucans, arabinogalactans, galactomannans and pectic substances. They are able to bind to dietary nutrients, hindering their digestion and absorption, thus decreasing the nutritional value of the feedstuffs of interest [28]. Commercial feed mixtures for broilers containing a high content of wheat are routinely supplemented with xylanase to eliminate the antinutritive effect of NSPs. Numerous studies have shown that xylanase in diets containing wheat decreases intestinal viscosity and increases nutrient digestibility and broiler performance [29–33]. Xylanase improves the immunity of chickens [34], reduces the unfavourable effect of *Salmonella typhimurium* infection [35] and increases the intestinal mucosal barrier in broiler chickens infected by *Clostridium perfringens* [36]. The use of xylanase was also successful in diets containing flaxseed [26].

This study was motivated by an effort to improve the welfare of broiler chickens. We assumed that the n-3 fatty acids contained in flaxseed had a positive effect on the quality of the chicken bones. Due to the presence of antinutritional substances in wheat-based mixed feed enriched in this way, the given effect may not be as pronounced. Therefore, the influence of the xylanase enzyme was assessed at the same time. Moreover, the effect of the addition of a single diet of xylanase to feed on the strength of bones in poultry is unclear in the literature. Therefore, the aim of our study was to determine the effects of thermostable xylanase in diets containing flaxseed on the performance of broiler chickens, digesta viscosity, nutrient retention, FA content in muscles, tibia strength and the interrelations among these factors.

## Materials and methods

### Cockerels, husbandry and diets

The experiment was conducted with 720 one-day-old Ross 308 cockerels. The cockerels were randomly assigned to four treatments with 12 replicate pens (15 cockerels per pen) in each treatment according to the contents of whole flaxseed (0 and 80 g/kg) and xylanase (0 and 0.1 g/kg) in the diet. The control diet did not contain flaxseed or xylanase (C). The second group was fed a diet supplemented with flaxseed (80 g/kg; F). The third group was fed a diet supplemented with xylanase (0.1 g/kg; X). The cockerels in the fourth group received a combination of xylanase and flaxseed (XF). Flaxseed was obtained from ANIMO CZ, Ltd. (Světice near Říčany, Czech Republic) and contained 202.0 g/kg crude protein, 386.5 g/kg ether extract and 254.0 g/kg crude fibre. The dosage of flaxseed was chosen based on our previous studies, where a significant effect of similar doses of flaxseed on the tibia quality of hens [37] and enzymatic activity in the digestive tract of broiler chickens [38] was recorded. Xylanase Nutrizyme® XY (Sunson Industry Group Co., Ltd., Beijing, China) is a highly concentrated xylanase preparation produced by submerged fermentation of *Trichoderma reesei*. This xylanase is thermostable and active over a wide pH range. The enzyme activity of xylanase was ≥ 15,000 U/g. The unit definition is as follows: 1 unit of xylanase equals the amount of enzyme that degrades 5 mg/ml xylan to release 1 μmol reducing sugar (xylose) in 1 min at 37°C at pH 5.5. In the case of xylanase supplementation, this is the dose normally recommended by the manufacturer. The diets contained 40% wheat. A balanced metabolizable energy concentration in the diet was achieved by adjusting the amount of added soybean oil. Flaxseed was not processed by grinding or extrusion. The results of the composition and nutrient analyses of the diets and flaxseed are shown in Tables 1–3. Throughout the experiment, chickens from individual groups received one mixed feed, which guarantees the invariability of the influence of this factor on the results. Feed and water were provided *ad libitum*.

**Table 1:** Composition and nutrient contents of the basal diet^a^.

Ingredient (g/kg)	C	F
**Wheat**	408.0	399.0
**Corn**	179.0	162.0
**Soybean meal**	328.0	294.0
**Flaxseed**	0.0	80.0
**Soybean oil**	44.2	21.0
**Calcium dihydrogen phosphate**	15.0	15.2
**Limestone**	13.5	13.8
**Sodium chloride**	2.0	2.0
**Sodium bicarbonate**	3.0	3.0
**L-Lysine**	0.8	2.1
**DL-Methionine**	1.4	2.0
**L-Threonine**	0.1	0.9
**Vitamin–mineral premix** ^**b**^	5.0	5.0
**Analysed nutrient content (g/kg)**		
**Dry matter**	892	896
**Crude protein**	208	206
**Ether extract**	59.8	60.4
**AME**_**N**_ **(MJ/kg) by calculation**	11.6	11.8
**Ca**	9.0	9.0
**Available P**	4.5	4.5
**Soluble NSPs**	21.5	37.6
**Insoluble NSPs**	82.7	85.9
**Total NSPs**	104.2	122.5

^a^Experimental mixed feeds were supplemented with xylanase (X) at 0 and 0.1 g/kg in the form of vitamin–mineral premix.

^b^Vitamin–mineral premix provided per kg of diet: retinyl acetate 3.6 mg; cholecalciferol 13 μg; niacin 40 mg; α-tocopheryl acetate 30 mg; menadione 3 mg; thiamine 3 mg; riboflavin 5 mg; pyridoxine 4 mg; cyanocobalamin 40 μg; calcium pantothenate 12 mg; biotin 0.15 mg; folic acid 1.5 mg; choline chloride 250 mg; ethoxyquin 100 mg; iron 50 mg; copper 12 mg; iodine 1 mg; manganese 80 mg; zinc 60 mg; selenium 0.2 mg.

AME_N_, nitrogen-corrected apparent metabolizable energy; Ca, calcium; P, phosphorus; NSPs, nonstarch polysaccharides.

**Table 2:** Concentrations of fatty acids in the diet (mg/100 g).

Diet	C	F	X	XF
Xylanase (X; g/kg)	0	0	0.1	0.1
Flaxseed (F; g/kg)	0	80	0	80
**SFA**	1085	1004	1043	1009
**MUFA**	1325	1212	1328	1424
**PUFA**	4275	5753	4207	5845
**n-3**	469	2150	454	2107
**n-6**	3806	3603	3753	3738
**n-6/n-3**	8.12	1.68	8.27	1.77

SFA, saturated fatty acids; MUFA, monounsaturated fatty acids; PUFA, polyunsaturated fatty acids.

**Table 3:** Activity of xylanase in mixed feed and liver (mg saccharide/g sample/h), n = 12/treatment.

Diet	C	F	X	XF		Probability		
Xylanase (X; g/kg)	0	0	0.1	0.1	SEM	X	F	X × F
Flaxseed (F; g/kg)	0	80	0	80				
**Mixed feed**	0.859	0.852	1.508	1.573				
**Liver**	0.463^b^	0.577^b^	0.728^b^	1.343^a^	0.0781	<0.001	0.001	0.021

^a,b^Means with different superscripts differ significantly.

SEM, standard error of the mean.

Cockerels were housed indoors in pens on wood shavings. The environmental conditions were maintained to meet the specific needs of the broilers. Heat was provided by a gas burner. During the first 3 days after hatching, the room temperature was set at 32°C and then gradually decreased to 20°C as the birds aged to 28 days. The lighting schedule for the first 7 days consisted of 23 hours of light and 1 hour of darkness, followed by a gradual reduction to 16 hours of light and an increase to 8 hours of darkness. Each pen was equipped with nipple drinkers and pan feeders. There was no restraint or suffering of the chickens during the experiment. The health status of cockerels was checked twice a day during the experiment based on chicken activity, normal behaviour patterns (e.g. active feed and water intake, normal walking, wing stretching, energetic movements when distracted or calm and effortless breathing), voice, plumage quality, skin, stance and foot and limb formation. The procedures performed with the animals were in accordance with the Ethics Committee of the Central Commission for Animal Welfare at the Ministry of Agriculture of the Czech Republic (Prague, Czech Republic) and were conducted in accordance with Directive 2010/63/EU for animal experiments. The experimental protocol was approved by the Ethical Committee of the Institute of Animal Science (Prague-Uhříněves, Czech Republic), case number 04/2021.

To determine performance characteristics, feed intake per pen was monitored daily, and the feed conversion ratio (FCR) was calculated by dividing the total feed intake by the overall weight gain over a 35-day period. The weights of the broilers were determined at the beginning of the experiment (Day 0), on the 14th day of age and at the end of the experiment (Day 35).

At 28 days of age, 12 chickens (1 chicken from each replicate, based on average body weight) were selected from each dietary treatment group and individually placed into cages to determine the total tract nutrient retention. The balance period lasted seven days. During the balance period, feed consumption and the amount of excreta produced were monitored daily. The excreta were collected and lyophilized prior to analysis. When the cockerels reached 35 days of age, 12 individuals were chosen from each dietary treatment group with a selection criterion based on average body weight (specifically, 1 cockerel from each of the 12 replicates). These selected cockerels were subsequently humanely slaughtered in certified slaughterhouses (for electrical stunning and exsanguination) without analgesia or anaesthesia. After the slaughter process, the carcasses were subjected to a 24-hour cooling period. Following this cooling period, the breast muscle, tibia, liver and ileum were dissected from the carcasses to obtain samples for subsequent chemical analyses.

### Nutrient analyses

Nutrient analyses (dry matter, crude fat, crude protein, crude fibre and ash) of the diets, excreta, breast meat and liver were conducted in accordance with methods established by the Association of Official Analytical Chemists (2005) [39], as stated in the study of Skřivan et al. (2020) [40]. The total phosphorus (P) content was determined using vanadate-molybdate reagent according to method No. 965.17 [39]. The capillary isotachophoretic method described by Dušková et al. (2001) [41] was utilized to determine the phytate P concentration in the diet. The Ca content was quantified by atomic absorption spectrometry using a Solaar M6 apparatus (TJA Solutions, Cambridge, UK).

### Xylanase activity

Xylanase (EC 3.2.1.32) activity in mixed feed and liver was assayed according to Taubner et al. (2023) [38] using a suitable substrate (xylan; Merck Life Science, Prague, Czech Republic) at a concentration of 4 mg/ml. The amount of reducing sugars in the supernatant was determined using a spectrophotometric method with copper and Nelson reagents [42]. The enzymatic activity was expressed as mg sugar/h per g of sample.

### Fatty acid profiles

The FA profiles of the flaxseed and breast meat were determined after chloroform–methanol extraction of the total lipids [43]. The alkaline trans-methylation of the FAs was performed as described by Raes et al. (2003) [44]. The fatty acid methyl esters (FAMEs) were determined by gas chromatography using an HP 6890 chromatograph (Agilent Technologies, Inc.) with a programmed 60 m DB-23 capillary column (150–230°C) and a flame-ionization detector. The FAs were identified by their retention times compared with those of standards: PUFA 1, PUFA 2, PUFA 3 and a 37-component FAME mixture (Supelco, Bellefonte, PA, USA).

### pH and viscosity of ileal digesta

The ileal digesta was collected in separate test tubes immediately after slaughtering the chickens and vortexed to obtain a homogenous content. The pH of the ileal digesta was measured with a digital portable pH meter (70, XS Instruments, Carpi, Italy). Approximately 15 ml of each sample was placed in centrifuge tubes and centrifuged at 3000 × g for 45 min. The viscosity of the supernatants was measured in triplicate with a Brookfield LDV-II+ PRO Digital Viscometer (Brookfield Engineering Laboratories, Inc., Stoughton, MA, USA). The measurement results are expressed in centipoise (cP) units.

### Bone quality characteristics

The tibia was used as a model bone to determine bone strength. Each left tibia bone was removed from the carcasses and thoroughly cleaned. The force required to break the tibia was subsequently assessed using an Instron 3342 device (Instron Worldwide Headquarters Norwood Ave, MA, USA). The equipment was equipped with a 50-kilogram load cell and operated at a crosshead speed of 50 mm per minute. Each bone was positioned on a span measuring 5.75 cm for testing. The ash content of the right tibia was determined. Each tibia was cleaned, boiled for 2 hours and again cleaned of tissue. The ash content in the tibia was determined after heating in a muffle furnace at 600°C for 24 hours.

### Statistical analyses

The data from the experiment were analysed by two-way analysis of variance (ANOVA) with a general linear model procedure in Statistical Analysis Software (Version 9.3, 2003) [45]. Differences between the groups were determined using the Duncan test. The main effects were the xylanase content (X), the flaxseed content (F) and the interaction between these two factors (X × F). The pen was the experimental unit (n = 12). The results in the tables are presented as the means and standard errors of the means (SEMs). Differences were considered significant at p ≤ 0.05.

The model used for the analysis was as follows:

Yijk=μ+αi+βj+γij+eijk

where Yijk represents the trait value; μ represents the overall mean; αi represents the effect of xylanase content in the diet (i = 1 and 2; 0 and 0.1 g/kg); βj represents the effect of the flaxseed content in the diet (j = 1 and 2; 0 and 80 g/kg); γij represents the interaction effect between these two factors; and eijk represents the random residual error.

## Results

### Xylanase activity

The highest activity of xylanase in the liver was found in chickens fed flaxseed with xylanase (p = 0.021) compared with that of the other groups, as shown in Table 3. The activities of xylanase in the diet and liver were strongly correlated with the amount of supplemented enzyme. The increases in activity by 77% and 57% (dietary treatment X vs. treatment C) are in agreement with the increases in the amounts of fat in the liver and breast muscles. These findings provide evidence that xylanase positively influences lipogenesis and affects the FA composition of meat.

### Performance characteristics

The addition of xylanase did not affect the weight of the chickens at the age of 14 days or their final weight (Table 4). Xylanase alone and in combination with flaxseed significantly decreased the feed intake (p = 0.050) and feed conversion ratio (p = 0.030) compared with those of the other groups. We can suppose that xylanase improved the health of chickens because the mortality of chickens significantly decreased (p = 0.05).

**Table 4:** Performance characteristics of broilers.

Diet	C	F	X	XF		Probability		
Xylanase (X; g/kg)	0	0	0.1	0.1	SEM	X	F	X × F
Flaxseed (F; g/kg)	0	80	0	80				
**Body weight (Day 0; g)**	37.5	36.5	36.9	35.7	0.19	NS	NS	NS
**Body weight (Day 14; g)**	370	378	373	387	3.7	NS	NS	NS
**Body weight (Day 35; g)**	2135	2150	2140	2169	19.1	NS	NS	NS
**Feed intake (g/bird per day)**	87.2^a^	89.1^a^	80.2^c^	83.4^b^	0.32	<0.001	NS	0.050
**FCR (kg/kg)**	1.46^a^	1.48^a^	1.33^c^	1.37^b^	0.08	<0.001	NS	0.030
**Mortality (%)**	3.9	2.6	1.3	2.0	0.09	0.050	NS	NS

^a-c^Means with different superscripts differ significantly; n = 12/treatment.

NS, not significant; SEM, standard error of the mean; FCR, feed conversion ratio.

### Nutrient retention

As shown in Table 5, a significant interaction effect of xylanase and flaxseed in the diet was detected in the retention of nitrogen-free extract (p = 0.011) and ash (p = 0.007). The control group presented decreased ash retention, and nitrogen-free extract retention was the lowest in broilers fed the diet with flaxseed. Xylanase significantly increased the retention of all the tested nutrients (p = 0.010 - < 0.001), except for crude fibre. Flaxseed supplementation did not influence performance but decreased the retention of dry matter (p = 0.016), crude protein (p = 0.012), organic matter (p = 0.016), and nitrogen-free extract (p = 0.008).

**Table 5:** Nutrient retention in broilers (g/kg/day).

Diet	C	F	X	XF	SEM	Probability		
Xylanase (X; g/kg)	0	0	0.1	0.1		X	F	X × F
Flaxseed (F; g/kg)	0	80	0	80				
**Dry matter**	50.2	39.4	56.9	54.7	2.03	0.001	0.016	NS
**Crude protein**	8.5	7.1	11.2	10.2	0.45	<0.001	0.012	NS
**Ether extract**	4.69	4.25	5.49	5.33	0.177	0.005	NS	NS
**Crude fibre**	-	0.598	0.596	0.578	0.0073	NS	NS	NS
**Nitrogen-free extract**	36.9^a^	26.1^b^	37.8^a^	37.5^a^	1.49	0.004	0.008	0.011
**Organic matter**	49.3	38.0	55.1	53.2	2.00	0.001	0.016	NS
**Ash**	0.91^c^	1.32^b^	1.82^a^	1.54^ab^	0.099	<0.001	NS	0.007
**Ca**	1.02	0.91	1.21	1.22	0.038	<0.001	NS	NS
**P**	0.199	0.192	0.247	0.215	0.0076	0.010	NS	NS

^a-c^Means with different superscripts differ significantly; n = 12/treatment.

NS, not significant; SEM, standard error of the mean.

### Fat content and fatty acid composition

Xylanase significantly increased the fat content in the breast meat of broilers (p = 0.029) (Table 6) and the concentration of PUFAs in the lipids of meat (p = 0.002) (Table 7). The presence of antinutrients in flaxseed likely limited the deposition of ALA in the muscles of the chickens in group F because when xylanase was added to the diet with flaxseed, the ALA content of the meat doubled (p < 0.001). The antinutrients in flaxseed, e.g. NSPs, increase intestinal viscosity and hinder nutrient resorption. The highest content of n-3 FAs in breast meat (p = 0.006) was detected in chickens fed diets supplemented with xylanase combined with flaxseed. All three experimental groups had reduced n-6/n-3 PUFA ratios (p = 0.001). The lowest n-6/n-3 ratios were found in the groups fed only flaxseed and those fed flaxseed combined with xylanase. The significantly lowest concentration of monounsaturated fatty acids in meat (p = 0.031) was found after the addition of flaxseed to the diet, contrary to the other groups. The profiles of FAs in feeds and meat may be used to define and evaluate one of the factors positively affecting the strength of the tibia in this study.

**Table 6:** Fat contents (g/kg) in breast meat and liver.

Diet	C	F	X	XF	SEM	Probability		
Xylanase (X; g/kg)	0	0	0.1	0.1		X	F	X × F
Flaxseed (F; g/kg)	0	80	0	80				
**Breast meat**	10.6	9.5	12.1	12.9	0.55	0.029	NS	NS
**Liver**	42.6	40.3	54.2	55.8	2.14	0.019	NS	NS

NS, not significant; SEM, standard error of the mean; n = 12/treatment.

**Table 7:** Fatty acid composition (mg/100 g) of breast meat.

Diet	C	F	X	XF	SEM	Probability		
Xylanase (X; g/kg)	0	0	0.1	0.1		X	F	X × F
Flaxseed (F; g/kg)	0	80	0	80				
**6:0**	0.025	0.022	0.037	0.044	0.0020	<0.001	NS	NS
**8:0**	0.115	0.113	0.082	0.081	0.0041	<0.001	NS	NS
**10:0**	0.114^a^	0.070^b^	0.078^b^	0.074^b^	0.0044	0.027	0.001	0.006
**12:0**	0.261	0.236	0.142	0.148	0.0116	<0.001	NS	NS
**13:0**	0.069	0.057	0.072	0.062	0.0024	NS	0.028	NS
**14:0**	1.96	1.69	2.16	2.27	0.087	0.023	NS	NS
**14:1-n5**	0.293	0.194	0.369	0.352	0.0182	<0.001	NS	NS
**15:0**	0.533	0.448	0.547	0.591	0.0232	NS	NS	NS
**16:0**	108^a^	84^b^	107^a^	114^a^	3.9	0.049	NS	0.036
**16:1-n7**	8.73	6.87	11.90	10.04	0.511	0.001	0.032	NS
**17:0**	0.902	0.780	1.050	0.967	0.0373	0.022	NS	NS
**18:0**	50.6	46.2	54.1	49.1	1.46	NS	NS	NS
**18:1-n9**	113^a^	84^b^	113^a^	125^a^	4.7	0.015	NS	0.015
**18:1-n7**	11.6	10.1	11.6	12.1	0.40	NS	NS	NS
**18:2-n6 t**	0.093	0.078	0.163	0.183	0.0100	<0.001	NS	NS
**18:2-n6**	138	99	162	146	6.1	0.001	0.006	NS
**18:3-n6**	2.76^ab^	2.96^a^	3.23^a^	2.11^b^	0.153	NS	NS	0.026
**18:3-n3**	11.1^c^	35.1^b^	17.4^c^	69.5^a^	4.33	<0.001	<0.001	<0.001
**18:2 (9.11)**	0.258^c^	0.434^b^	0.413^b^	1.026^a^	0.0566	<0.001	<0.001	<0.001
**18:2 (10.12)**	0.043	0.044	0.055	0.060	0.0020	<0.001	NS	NS
**20:0**	0.394	0.381	0.558	0.497	0.0202	<0.001	NS	NS
**20:1-n9**	1.19	0.82	1.45	1.22	0.058	0.001	0.002	NS
**20:2-n6**	5.29^a^	3.01^c^	5.06^ab^	4.26^b^	0.214	NS	<0.001	0.019
**21:0**	0.142	0.146	0.216	0.201	0.0083	<0.001	NS	NS
**20:3-n6**	4.39	3.11	3.50	3.19	0.160	NS	0.008	NS
**20:4-n6**	35.3	18.9	30.4	20.4	1.45	NS	<0.001	NS
**20:3-n3**	1.13^b^	2.57^a^	0.76^b^	2.80^a^	0.170	NS	<0.001	0.032
**20:4-n3**	0.202^b^	0.205^b^	0.193^b^	0.414^a^	0.0192	<0.001	<0.001	<0.001
**22:0**	0.095	0.094	0.130	0.151	0.0058	<0.001	NS	NS
**20:5-n3**	1.11	6.03	1.43	6.71	0.474	0.042	<0.001	NS
**22:1-n9**	0.104^bc^	0.097^c^	0.119^b^	0.154^a^	0.0057	<0.001	NS	0.019
**23:0**	0.068	0.058	0.078	0.090	0.0037	0.003	NS	NS
**24:0**	0.082	0.069	0.072	0.081	0.0031	NS	NS	NS
**22:5-n3**	6.55	15.36	6.91	12.81	0.791	NS	<0.001	NS
**24:1-n9**	0.108	0.096	0.112	0.109	0.0039	NS	NS	NS
**22:6-n3**	4.21	6.29	2.94	5.04	0.267	0.001	<0.001	NS
**SFA**	163	135	166	168	5.3	NS	NS	NS
**MUFA**	135^a^	102^b^	138^a^	149^a^	5.6	0.014	NS	0.031
**PUFA**	210	193	235	274	9.2	0.002	NS	NS
**n3**	24.3^c^	65.5^b^	29.6^c^	97.2^a^	5.70	<0.001	<0.001	0.006
**n6**	186	127	205	176	7.6	0.006	0.001	NS
**n6/n3**	7.66^a^	1.96^c^	6.96^b^	1.81^c^	0.491	<0.001	<0.001	0.001

^a-c^Means with different superscripts differ significantly; n = 12/treatment.

NS, not significant; SEM, standard error of the mean; SFA, saturated fatty acids; MUFA, monounsaturated fatty acids; PUFA, polyunsaturated fatty acids.

### Digesta viscosity and pH

A significant interaction effect of flaxseed and xylanase enzyme was noted for the viscosity (p = 0.043) and pH (p = 0.010) of the ileal digesta (Table 8). The highest values of both indicators were found in chickens fed a diet with flaxseed and the lowest values were found in chickens with xylanase in the diet. Compared with the C treatment, the flaxseed in feed mixture F increased the digesta viscosity in the ileum by 55%. Dietary addition of xylanase suppressed viscosity by 35% compared with that of C and 58% compared with that of F.

**Table 8:** Digesta viscosity and pH in the ileum.

Diet	C	F	X	XF	SEM	Probability		
Xylanase (X; g/kg)	0	0	0.1	0.1		X	F	X × F
Flaxseed (F; g/kg)	0	80	0	80				
**Viscosity (cP)**	3.82^b^	5.94^a^	2.50^c^	3.06^bc^	0.160	0.015	0.012	0.043
**pH**	6.23^b^	6.81^a^	5.94^c^	6.32^b^	0.090	0.001	<0.001	0.010

^a-c^Means with different superscripts differ significantly; n = 12/treatment.

SEM, standard error of the mean.

### Breaking strength and ash content of the tibia

As shown in Table 9, compared with the control diet, alone flaxseed and xylanase in the diet increased the strength of the tibia (p = 0.030). The ash concentration in the tibia was the highest after supplementation of the basal diet with flaxseed and flaxseed supplemented with the enzyme xylanase (p = 0.009).

**Table 9:** Breaking strength and ash contents in the tibia.

Diet	C	F	X	XF	SEM	Probability		
Xylanase (X; g/kg)	0	0	0.1	0.1		X	F	X × F
Flaxseed (F; g/kg)	0	80	0	80				
**Strength (N)**	332^b^	389^a^	385^a^	366^ab^	8.1	NS	0.010	0.030
**Ash (g/kg)**	385^c^	443^a^	401^b^	437^a^	17.3	<0.001	<0.001	0.009

^a-c^Means with different superscripts differ significantly; n = 12/treatment.

NS, not significant; SEM, standard error of the mean.

## Discussion

In the present study, a diet containing wheat, corn and soybean meal was supplemented with flaxseed to increase the n-3 PUFA content. Flaxseed is an interesting component of poultry feed mixtures, but it can have a negative effect on performance parameters [14, 15]. Feeding ground flaxseed in excess of 7.5% dry matter may reduce growth and body weight, resulting in poor feed conversion efficiency. This phenomenon is related to the relatively large amounts of dietary fibre or NSP in flaxseeds [16]. The concentration of soluble NSP in flaxseed is up to twice as high as that in cereals. A significant part of the fibre dissolves in water, forming highly viscous solutions [17]. Additionally, water-soluble mucilage present in the hull fraction of flaxseed significantly increases the intestinal viscosity [18, 19]. The viscosity of jejunal digesta is correlated with the digestibility of nutrients in chickens. The removal of the mucilage layer of flaxseed increased the digestibility of energy and fat, which confirms the effect of NSPs [46]. The high viscosity of digesta may limit the digestion and absorption of nutrients in young birds [20]. Young birds are more sensitive to high intestinal viscosity; thus, flax meal should be avoided in the diets of young birds. Flaxseed also contains other antinutritional components, including cyanogenic glycosides or the pyridoxine antagonist linatine. Pelleting reduces the danger of poisoning by cyanogenic glycosides because of the combined effect of heat and pressure. Pelleting modifies the physical structure of seeds and increases the availability of lipids to intestinal lipases [47]. In our study, as well as in the work of Popescu et al. (2021) [48], no significant influence of the addition of flaxseed to the diet on performance indicators was recorded. Popescu et al. (2021) [48] even reported that dietary supplementation with flaxseed meal may increase the intestinal health status of poultry.

The amount of xylanase added to experimental diets varies greatly among individual authors, not only according to the microbial origin of the enzyme. Thus, Van Hoeck et al. (2021) [49] added 30,000–90,000 U/kg xylanase. Luo et al. (2009) [50] added only 500–5000 U/kg xylanase, and the greatest effect was achieved with the lowest additions of 500 and 1000 U/kg xylanase. According to the manufacturer’s recommendation, 1500 U/kg xylanase was used in our study, which, according to Luo et al. (2009) [50], was close to the most effective amount of xylanase added.

The present study and similar studies performed earlier have shown that the addition of xylanase to poultry feeds improved the nutritive value of wheat [22, 33, 49]. In agreement with previous studies, Van Hoeck et al. (2021) [49] concluded that the beneficial effects of xylanase occur primarily through a decrease in the viscosity of the intestinal environment and secondarily through the release of fermentable sugars. Xylanase decreased feed intake and feed conversion without affecting the growth of broilers in the present study. Al-Qahtani et al. (2021) [51] reported lower feed consumption and feed conversion ratio in chickens fed xylanase. However, Apperson and Cherian (2017) [25] did not find an effect of the flax level or enzyme supplementation on production performance parameters such as final body weight, feed consumption, and average daily gain. Exogenous enzymes might provide a greater benefit to newly hatched chicks as they transition from a lipid to a carbohydrate diet while their immune system and digestive tracts are developing [52]. This is also evidenced by the positive effect of xylanase on nutrient retention in our work. Moreover, Zhang et al. (2014) [53] reported that the supplementation of xylanase in wheat-based diets cuts the arabinoxylan backbone into small fragments (mainly arabinose and xylose) in the ileum, jejunum and duodenum and enhances the digestibility of nutrients by decreasing digesta viscosity. This is also evident from the study conducted by Liu and Kim (2017) [54]. Xylanase supplementation improves the performance, nutrient digestibility, and digestive function of broiler chickens [55]. On the other hand, no effect of xylanase inclusion on the performance or nutrient digestibility of laying hens was found in the study of Lei et al. (2018) [56]. In addition, a synergistic effect of xylanase and flaxseed on xylanase activity was detected in the liver of chickens in the present study. This finding is consistent with the results of a study by Taubner et al. (2023) [38], who reported that flaxseed supported enzyme activity in the gastrointestinal tract in broilers.

A pronounced effect of flaxseed supplementation was found in the fatty acid profile of the breast meat. Notably, the effect of flaxseed on the n-6/n-3 PUFA ratio in meat lipids. Based on epidemiological and clinical studies, Wijendran and Hayes (2004) [57] recommended an n-6/n-3 PUFA ratio of approximately 6:1. The high value of this ratio has been criticized by nutritionists; thus, its reduction by flaxseed is desirable. Additionally, xylanase increased the PUFA content in the breast meat of chickens in the present study. This finding is consistent with the work of Papadopoulos et al. (2022) [58], who reported that xylanase supplementation at a relatively high level (90,000 U/g) improved polyunsaturated and reduced monounsaturated egg yolk FA content. The change in the fatty acid profile of poultry products after the administration of xylanase is probably related to the reduction in intestinal viscosity, which allows better digestion and absorption of fats and other substances. Moreover, xylanase enhances fat digestion by reducing bile salt deconjugation [59]. In addition, this may be due to the depolymerization of the cell wall polysaccharides after xylanase supplementation, which resulted in more oil release and thus more oil exposure to digestive enzymes [14].

Interesting results have been reported for the bone health of chickens. More specifically, the group fed flaxseeds and xylanase, but not their combination (although an increase was observed), presented significant differences in tibia fracture strength. These results are due to both the lipid content of flaxseed and the active role of xylanase in bone metabolism. In the first case, as reported in the literature, n-3, particularly ALA, contained in flaxseeds, is able to improve bone health through its converting forms (eicosapentaenoic acid and DHA). These FAs are able to act directly on bone marrow-derived macrophages and decrease osteoclastogenesis, osteoclast maturation, and bone resorption. Alpha-linolenic acid preserves bone mass by increasing the expression of key transcription factors, such as osteocalcin, which allows the differentiation of prosteoblasts into mature osteoblasts and thus bone formation [60]. The positive influence of n-3 PUFAs has also been confirmed in the literature by further studies conducted by Tartlon et al. (2013) [4] and Toscano et al. (2015) [61]. As previously reported, the xylanase group also presented improved bone health, most likely due to increased mineral substance retention after xylanase supplementation. This result is confirmed in the study conducted by Muszynski et al. (2020) [62]. More precisely, the authors demonstrated how xylanase supplementation was able to influence the carbonate/phosphate ratio and crystallinity index in laying hens. Moreover, xylanase increases the calcium content in the tibia, as well as the contents of several trace elements (magnesium, manganese and chromium) crucial for improving bone health [62, 63]. Furthermore, the addition of xylanase promoted mineral absorption by increasing calcium homeostasis, resulting in improved bone quality [62]. This result is a key point in poultry farming, especially following the ban of battery cages in the European Union, which resulted in increased bone fractures and mortality in chickens housed in barns or free-range systems.

## Conclusions

Thermostable xylanase in a wheat-type diet reduced ileal viscosity and improved the utilization of nutrients, including mineral substances, thus increasing the performance characteristics of chickens and tibia strength. Xylanase addition to a flaxseed diet can reduce the antinutritional effect of flaxseed by reducing the viscosity of the digesta. Xylanase in the flaxseed diet increased the content of health-promoting n-3 PUFAs in the meat and retention of nitrogen-free extract and decreased the feed intake and feed conversion ratio compared with those of flaxseed alone in the diet. These results demonstrate the importance of using commercial enzymes to improve diets and feed ingredients that can work synergistically to ensure animal health and high performance. For this reason, scientific research has the task of further investigating this issue, not only by identifying the best flaxseed concentrations in broiler diets but also through the enhancement of additional ingredients that can ensure food/feed safety, animal health and environmental protection.
